# Detection of Peste des Petits Ruminants Viral RNA in Fecal Samples of Goats after an Outbreak in Punjab Province of Pakistan: A Longitudinal Study

**DOI:** 10.1155/2016/1486824

**Published:** 2016-08-15

**Authors:** Riasat Wasee Ullah, Aamer Bin Zahur, Asma Latif, Javid Iqbal Dasti, Hamid Irshad, Muhammad Afzal, Tahir Rasheed, Adnan Rashid Malik, Zafar-ul-Ahsan Qureshi

**Affiliations:** ^1^Veterinary Research Institute, Zarar Shaheed Road, Lahore Cantt 54810, Pakistan; ^2^Department of Microbiology, Faculty of Biological Sciences, Quaid-i-Azam University, Islamabad 45320, Pakistan; ^3^Animal Health Research Laboratories, Animal Sciences Institute, National Agricultural Research Centre, Islamabad 44000, Pakistan; ^4^Progressive Control of PPR in Pakistan, FAO-UN, Islamabad 44000, Pakistan; ^5^Department of Animal Husbandry, Azad Government of the State of Jammu and Kashmir, Muzzafarabad 13100, Pakistan

## Abstract

Peste des petits ruminants (PPR) is a highly contagious viral disease of domestic and wild small ruminants and thus has serious socioeconomic implications. In Pakistan, during the year 2012-2013, estimated losses due to PPR were worth Rs. 31.51 billions. Close contact between infected and susceptible animals is an important route of transmission of PPR. Therefore, carrier animals play an important role in unnoticed transmission of PPR. The objective of the study was to investigate the detection of PPR virus in goats recovered from PPR. A suspected PPR outbreak was investigated and confirmed as PPR after analysing appropriate samples collected from infected animals using rRT-PCR. A longitudinal study was conducted over the period of 16 weeks to ascertain the detection of PPR virus (PPRV) in faecal samples of recovered goats. Ninety-six (96) faecal samples from each sampling were collected at 4, 8, 12, and 16 weeks after the outbreak. Faecal samples were analysed using rRT-PCR. Of 96 from each sampling a total of 46, 37, 29, and 25 samples were positive for PPR viral genome at 4, 8, 12, and 16 weeks, respectively, after recovery. Attempts were made for the isolation of PPR virus on Vero cells, but results were negative. These results indicated the detection of PPR viral RNA up to 16 weeks after infection. Therefore, these results may help in the future epidemiology of PPR virus shedding and possible role as source of silent infection for healthy animals especially when there is no history of any outbreak in nearby flock or area.

## 1. Introduction

Peste des petits ruminants (PPR) is a highly contagious viral disease of domestic and wild small ruminants caused by PPR virus (PPRV) of family Paramyxoviridae [1]. The disease is associated with high morbidity (100%) and mortality (up to 90%) [2]. PPR is also classified as transboundary animal disease (TAD) [3]. The typical form of PPR is associated with anorexia, pyrexia, ulceration, necrosis of mucous membranes, sores in mouth, mucopurulent nasal and ocular discharges, pneumonia, inflammation of the gastrointestinal tract (GIT), and diarrhea [4–6]. Animals of all ages are susceptible to the disease. However, the disease is more fatal in kids and lambs. A close contact with infected animals is considered an important mean of transmission of disease [7].

Optimizations of advanced methods for the detection of PPRV are important for large scale surveillance studies especially to investigate carrier state of the animals. So far, very few reports have been documented in this regard. However, following the acute phase of PPR in small ruminants some animals may experience a long asymptomatic persistent stage. PPR viral RNA detection has been reported for 11 weeks after recovery and in some animals even up to 12 weeks after recovery using haemagglutination (HA) test [8]. Furthermore, it has also been reported that animals infected with PPR virus are incubatory carriers and virus shedding was detected in their secretions and excretions 2-3 days prior to the onset of the clinical disease [9]. Under given conditions these carriers may contribute to the unnoticed transmission of PPR virus at high risk areas like weekly livestock markets and during communal grazing at pastures. Such asymptomatic carriers are threat to livestock farmers and may contribute to transboundary episode of the disease. This suggests the need to carry out studies to understand the persistence and carrier state of small ruminants and further transmission of the disease. There are no systematic studies reported about persistence and carrier state of PPR. We report a longitudinal study that has the objective to determine the duration of detection of PPR viral RNA in the faecal samples of animals recovered from PPR.

## 2. Materials and Methods

### 2.1. Outbreak Investigation and Collection of Samples

A suspected outbreak of PPR was investigated in an organised goat farm in suburban area of Lahore district, Pakistan, on 20 November 2012. The affected animals were examined for the presence of clinical signs specific to PPR virus infection. The temperature and clinical signs were recorded on prescribed proforma. The outbreak control measures were implemented and symptomatic therapeutic interventions were advised to the farmer. Structured epidemiological investigations were conducted to determine the most likely source of PPR virus transmission. Information regarding previous history of the disease at the farm, vaccination status of the flock, flock size, number of affected animals, number of dead animals, and history of a PPR outbreak in the nearby farm/area was recorded on a prescribed proforma.

Necropsy of recently dead animals was performed. The carcasses were examined for the evidence of discharges (ocular and nasal), diarrhea, and pneumonia. Gross pathological lesions in GIT and respiratory tract were recorded.

Ocular, nasal, and oral swabs and faecal samples were collected from live animals. Ocular swabs were collected by inserting a sterile swab (BD sterile swab) beneath the conjunctiva and swirling it so that the ocular secretions may adhere to the swab. The oral and nasal swabs were collected by inserting a sterile swab (BD sterile swab) deep into the oral and nasal cavity. Faecal samples were collected directly from rectum of clinically affected goats in polyethylene zipper bags using sterile gloves. The gloves were changed after collecting each sample.

The tissue samples including lungs, liver, spleen, lymph nodes (mesenteric and bronchial), kidneys, and intestine were collected from dead animals. Each sample from donor animal was given a unique identification number with date. All the samples were transferred to laboratory in cold conditions. The swabs, tissues, and fecal and sera samples were stored at −70°C till further analysis.

### 2.2. Analysis of Samples

Oral, ocular, and nasal swabs were processed for analysis. The cotton area of the swab was separated out gently from swab stick with the help of sterile forceps and scissor. Swab was put into a sterile Eppendorf tube containing 1.5 mL of sterile phosphate buffer saline (PBS; 0.01 M pH 7.4). The swab was completely squeezed in 1.5 mL Eppendorf tube and centrifuged at 10,000 RPM for 3–5 minutes at 4°C. Supernatant was collected and stored at −70°C till further analysis.

The tissue samples were processed by making approximately 10% homogenate of infected tissues in sterile PBS (0.01 M pH 7.4). The homogenate was centrifuged at 10,000 RPM for 3–5 minutes at 4°C in a 1.5 mL Eppendorf tube. The supernatant was collected and stored at −70°C till further analysis.

RNA was extracted from all the samples using RNeasy kit (Qiagen GmbH, Hilden, Germany). A negative control was also included for detection of possible contamination during extraction. The extraction of RNA was performed according to manufacturer instructions. Briefly, 560 *μ*L of lysis buffer and 700 *μ*L of 70% ethanol have been added to 140 *μ*L of tested sample and allowed to spin in spin filtered column for one minute. For washing, wash buffer and buffer RPE are added and allowed to spin. To elute the extracted RNA, 40 *μ*L of RNA's free water was used. The extracted RNA was placed at −20°C until further use. The quantity and purity of extracted RNA were determined using NanoDrop (NanoDrop 1000, Thermo Scientific, Wilmington, DE, USA) [10].

Each sample was analysed for the presence of PPR virus specific genome using reverse transcriptase real time polymerase chain reaction (rRT-PCR) [11]. rRT-PCR was done using core reagent kit (TaqMan EZ-RT-PCR Core Reagent), sequence specific primers, and probes described by [11]. Briefly, 5.0 *μ*L of TaqMan EZ buffer, 2.5 *μ*L of 25 mM Mn (OAc)_2_, 03 *μ*L of dNTPs, 01 *μ*L of forward primer, 01 *μ*L of reverse primer and 01 *μ*L of probe, 01 *μ*L of rTh DNA polymerase, and 10.5 *μ*L of nuclease free water were added to make the volume 25 *μ*L. Master mix (22.5 *μ*L) and 2.5 *μ*L of RNA template were added to the wells of optical 96-well reaction plate (MicroAmp*™* N801-0560). The plate was covered with adhesive film (MicroAmp) and spun in refrigerated centrifuge at 2500 rpm for 1 minute.

The rRT-PCR was performed using ABI7500 real time PCR system (Applied Biosystems) and ABI prism SDS software, an initial reverse transcription temperature at 45°C for 30 min, followed by reverse transcriptase inactivation and DNA polymerase activation at 95°C for 5 min, and then 50 cycles of 15 s at 94°C and 30 s at 60°C. The reporter dye (FAM) signal was measured against the internal reference dye (ROX) signal to normalize the signals for non-PCR-related fluorescence fluctuations that occur from well to well. The data were collected at the annealing step of each cycle and the threshold cycle (Ct) for each sample was calculated by determining the point at which the fluorescence exceeded the threshold limit.

### 2.3. Study Design

The animals positive for PPR were followed for the period of four months from 20 November 2012 to 20 March 2013. Sera and fecal samples were collected from recovered animals at 4, 8, 12, and 16 weeks after outbreak.

The sera samples were analysed for the presence of PPRV specific antibodies using anti- nucleocapsid (N) monoclonal antibody (MAb) based competitive ELISA (c-ELISA) [12]. The faecal samples were analysed using rRT-PCR [11].

## 3. Results

### 3.1. Epidemiological Observation

The animals were raised on a semiextensive system where the animals were taken out for grazing in the morning and supplemented with a concentrate stall feeding in the evening for fattening. The flock consisted of 140 goats with age ranging between 10 and 18 months. The flock had history of introduction of five new animals from a nearby livestock market. None of the animals had a history of vaccination against PPR. There was no outbreak of PPR in the nearby area/village. The morbidity rate was 100%. However, 44 out of 140 animals died during the outbreak with a mortality rate of 31.42%.

### 3.2. Clinical Picture

The clinical examination of the affected animals revealed high fever ranging between 39°C and 42°C, conjunctivitis, mucopurulent nasal and discharges along with depression, anorexia, swollen lips, cough, and diarrhoea. The affected animals showed signs of severe dehydration and their hind quarters were soiled with diarrhoea material. The mouth lesions were found in all the affected animals with red raw areas on inner side of the lips, lower gums, and necrosis on the dorsal surface of the tongue.

### 3.3. Postmortem Findings

The postmortem examination of *n* = 3 recently dead animals was conducted. On external appearance the carcasses were found to have evidence of dehydration with sunken eyes, rough/dry skin, and hind quarter soiled with diarrhoea material. The internal examination revealed pneumonic lungs with haemorrhages on mucosal surfaces of rumen, abomasum, and large intestine (caecum and colon). Haemorrhages were also observed on liver and kidneys of one animal. The body lymph nodes were inflamed and swollen particularly the mesenteric lymph nodes.

For laboratory confirmation of PPR a total of 29 swabs and 33 faecal and 12 tissue (lungs = 3, liver = 2, lymph nodes = 4, spleen = 2, and kidney = 1) samples were collected. Sixteen (55.17%) swabs and 28 (84%) faecal and 10 (83%) tissue samples were found positive for PPRV genome using rRT-PCR. These results confirmed the outbreak of PPR in the flock.

### 3.4. Fecal Samples

**Figure 1:**
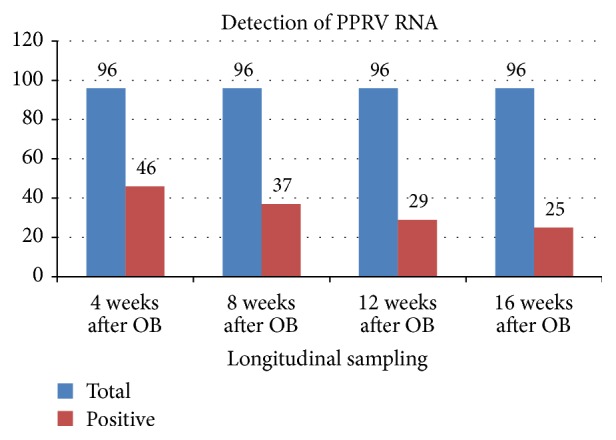
Monthwise detection of PPRV genome from fecal samples.

**Figure 2:**
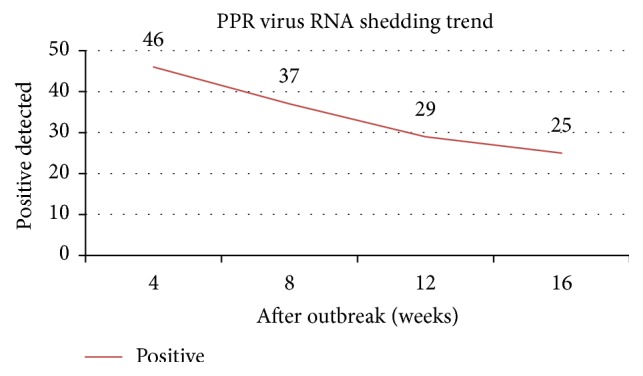
Decreasing trend of PPR viral RNA shedding up to 16 weeks after outbreak in recovered animals.

The samples were collected according to the method as described earlier. At each sampling *n* = 96 faecal samples were collected. RNA extraction and rRT-PCR were performed as mentioned earlier. At each sampling *n* = 96 faecal samples were collected from PPR recovered goats, out of which 46, 37, 29, and 25 samples were found positive for PPR viral genome at 4, 8, 12, and 16 weeks, respectively. These results indicated that small ruminants shed PPR viral genome up to four months (16 weeks) after clinical recovery from the disease (Figures 1 and 2).

## 4. Discussion

A PPR outbreak causing high morbidity and low mortality in a goat herd in Punjab was confirmed on the basis of clinical signs and various lab diagnostic tests. The animals recovered from the outbreak were monitored and their faecal samples were collected for a period of 16 weeks to determine the persistence of PPRV in faecal samples of recovered animals.

The results of the study indicated that introduction of new animals into the flock from weekly livestock markets resulted in the initiation of an outbreak in herd of goats under investigation. Similar mode of disease transmission has been reported previously by Asmar et al. [13] in Saudi Arabia where outbreaks in sheep were attributed to the introduction of new animals from livestock market. Under such conditions the newly introduced animals remained unaffected while rest of the flock experienced PPR virus infection. This may suggest that newly introduced animals may have experienced infection in the past and become carrier of the virus. PPR outbreaks have been reported in healthy herds after the introduction of newly purchased potentially incubating animals to the flocks [14].

During investigation typical clinical signs and symptoms of PPR virus infection were observed in infected animals. These include high fever up to 42°C, lesions in mouth, oral and nasal congestion, respiratory signs, and diarrhea leading to death of the animals. Similar findings were made in previous reports [9, 15, 16]. In this study it was observed that the morbidity rate was 100% while mortality rate was 31.42%. These findings are in complete concurrence with previous study which reported morbidity and mortality rates due to PPR ranging from 0 to 90% depending on the local husbandry practices, breed, age, and other factors [17]. The presence of PPR virus in current study was confirmed by clinical signs, postmortem examination, and rRT-PCR, while competitive ELISA (c-ELISA) was used for antibody detection.

The results of the study indicated detection of PPRV in goats recovered from PPR. However, the number of animals giving positive results decreased from 46 on 4th week after vaccination to 25 on 16th week after vaccination. Previous studies also reported the persistence of PPRV antigen in faecal samples of PPR recovered goats [8, 18]. However, the duration of detection of PPRV antigen in various studies was different. For example, Ezeibe et al. [8] reported detection of PPRV antigen using HA in faecal samples of recovered goats up to 12 weeks after recovery. Another study reported persistence of PPRV in faecal samples of recovered goats up to 30 days in vaccinated and 60 days in unvaccinated goats after recovery [18].

Various samples have been analysed using different diagnostic tests to understand the persistence of PPRV in animals recovered from PPR [6]. For example, a recent study reported the persistence of PPRV after challenge for 40 days in nasal, ocular, and oral swabs [19]. In contrast, another study reported the persistence of PPRV in faecal samples of recovered goats for 12 weeks. Secondly the aim of the study was to determine the duration of detection of PPR viral RNA in the faecal samples of animals recovered from PPR to understand the role of faecal samples through this route. Therefore, faecal samples appeared to be the sample of choice for persistence studies.

We used rRT-PCR for detection of PPRV in faecal samples of goats whereas previous studies used HA for detection of PPRV in faecal samples [8, 18]. rRT-PCR is considered a highly specific test for detection of PPRV [11]. However, a previous study evaluating HA for detection of PPRV in faecal samples of sheep and goats reported low specificity of HA for detection of PPRV compared to reverse transcriptase PCR [20]. Therefore, HA may not be considered a suitable test for detection of PPRV antigen in faecal samples of PPR recovered goats.

In conclusion, this study reported the detection of PPRV in faecal samples of PPR recovered goats using rRT-PCR up to 16th weeks after recovery indicating the possible role of PPR recovered goats in transmission of disease to in-contact healthy animals. However, a study should be carried out to isolate the virus from faecal samples positive for PPRV RNA to further elucidate the role of recovered animals in transmission of disease to healthy animals. Therefore, these results may help in studying the future epidemiology of PPR virus shedding and transmission of PPR virus by fecal material and possible role as source of silent infection for healthy animals especially when there is no history of any outbreak in nearby flock or area.
